# Health‐related quality of life (HRQoL) and associated factors in Bangladeshi adolescents during COVID‐19

**DOI:** 10.1002/hsr2.1927

**Published:** 2024-02-22

**Authors:** Afifa Anjum, Sabrina Mousum, Zubair Ahmed Ratan, Marium Salwa, Md Maruf H. Khan, Mohammad Tanvir Islam, S. M. Yasir Arafat, M. Atiqul Haque

**Affiliations:** ^1^ Department of Psychiatry University of Cambridge Cambridge UK; ^2^ Department of Public Health and Informatics Bangabandhu Sheikh Mujib Medical University Dhaka Bangladesh; ^3^ Department of Biomedical Engineering Khulna University of Engineering and Technology Khulna Bangladesh; ^4^ School of Health & Society, Faculty of The Arts, Social Sciences and Humanities University of Wollongong Wollongong New South Wales Australia; ^5^ Department of Internal Medicine Bangabandhu Sheikh Mujib Medical University Dhaka Bangladesh; ^6^ Department of Psychiatry Enam Medical College and Hospital Dhaka Bangladesh

**Keywords:** adolescents, Bangladesh, COVID‐19, HRQoL, rural, urban

## Abstract

**Background and Aims:**

The COVID‐19 pandemic and its accompanying countermeasures significantly disrupt the health‐related quality of life (HRQoL) of adolescents. We aimed to estimate the status and associated factors related to HRQoL of adolescents during the COVID‐19 pandemic from the community population of Bangladesh.

**Methods:**

This cross‐sectional study followed two‐stage sampling. From eight administrative divisions of Bangladesh, 2030 adolescents were enrolled. The KIDSCREEN‐10 index was used to measure the HRQoL of adolescents. In addition to this, adolescents' data on sociodemographics, mental well‐being, parenting style, insomnia, food insecurity, depression, anxiety and stress, resilient coping, screen‐based activity, and anthropometry were taken for finding out the factors associated with HRQoL of adolescents. The hierarchical multilinear regression was performed to assess the association.

**Results:**

More than 47% of adolescents were found to have moderate and high HRQoL, while 4.7% of adolescents experienced low HRQoL during data collection. Higher age (B: −0.671), having more siblings (B: −0.316), food insecurity (B: ‒2.010), depression (B: ‒0.321), anxiety (B: ‒0.362), and stress (B: ‒0.150) were found to have significantly negative associations with adolescents' HRQoL during the COVID‐19 pandemic. Whereas, positive parenting (B: 0.409), inconsistent parenting discipline (B: 0.266), good mental health (B: 5.662), resilient coping (B: 0.306) were found to have significant positive relationships.

**Conclusions:**

The findings from this study indicate that over 52% of the adolescents reported a moderate and lower level of HRQoL. In light of these results, it may be beneficial to prioritize interventions targeting psychological factors such as depression, anxiety, and stress.

## 1 BACKGROUND

### 1.1 Health‐related quality of life (HRQoL)

The notion of quality of life (QOL) is broad, complex, and multidimensional, and it is open to subjective interpretations, which typically encompass both positive and negative aspects of life.^1^ The World Health Organization (WHO) defines the HRQoL as “an individual's perception of their position in life in the context of the culture and value systems in which they live, and in relation to their goals, expectations, standards, and concerns.”^2^ According to Riley et al., the HRQoL assessment comprises assessing physical, psychological, and social well‐being at an early age based on an individual's evolutionary growth within a cultural context.^3^ The HRQoL assessment also takes into account the capacity for full involvement in various activities, as well as the physical, social, and psychosocial functions that are appropriate for early ages.^3^ Children and adolescents with low HRQoL have the probability of abnormal development and consequently mature into unhealthy adults.^3^ Since individual and contextual factors influence HRQoL, adopting an ecological perspective is important for improving the HRQoL of children and adolescents. This adoption might be the complex interaction of different systems that influence the values, beliefs, and, ultimately, the QOL of children and adolescents.^4^

### 1.2 COVID‐19, HRQoL and adolescents

The COVID‐19 pandemic has impacted the lives of adolescents from different aspects. For example, different studies have stated that during the COVID‐19 pandemic, the screen‐based recreational time of adolescents has increased.^5^, ^6^ As a result, sedentary behaviors and lifestyles among adolescents have increased in ways that have been linked to low to moderate HRQoL, as reported in earlier studies.^7^, ^8^ Additionally, coping with the current pandemic situation and compliance with the restrictions may worsen adolescents' mental health and HRQoL.^9^

The COVID‐19 pandemic has affected adolescent mental health, hence HRQoL, as mental health is a facet of HRQoL. Aside from the recognized risk factors for mental health problems in adolescents during the COVID‐19 pandemic, misinformation, high mortality, and resource and food insecurity have been linked.^10^ Xie et al. found that 23% of 2nd‐ to 6th‐grade students exhibited depressed symptoms, and 19% had anxiety symptoms.^11^ In another study, Zhou et al. found that among 12–18‐year‐olds, 44% had depressive symptoms, 37% had anxiety, and 31% had both.^12^ These mental health issues may persist throughout adulthood.^13^ These comorbid mental and physical health issues can worsen HRQoL in adolescence and adulthood.^14^, ^15^

### 1.3 Theoretical framework

Ravens‐Sieberer et al. stated that compared to adults, adolescents' HRQoL is quite a new aspect of research, warranting special guidelines from relevant authorities, such as the Mental Health Division of the WHO.^16^ They defined HRQoL must be “such measures should be age‐appropriate and child‐centred, preferably take into account self‐reporting, be usable independently of the health status and cross‐culturally, and should include both positive and negative aspects.”^17^ Apart from these, the instruments for measuring the HRQoL should meet the appropriate psychometric properties.^16^

HRQoL is generally regarded as a latent construct that cannot be directly observed. Regardless of the definition, it encompasses an individual's perception and judgment of their life from their subjective viewpoint, including their subjective well‐being and emotional state. These characteristics highlight the importance of self‐reporting when measuring QOL, particularly in the case of children and adolescents. It is essential to consider their developmental stage and the ongoing changes that occur over time, including shifts in their emotional perceptions.^16^

In line with this theoretical framework, our study aimed to investigate the HRQoL of adolescents during the unique circumstances of the COVID‐19 pandemic. We utilized the self‐reported KIDSCREEN‐10 questionnaire, recognizing that the pandemic introduced various challenges and altered perceptions. These changes, coupled with the subjective well‐being and developmental transitions of adolescents living in both urban and rural areas of Bangladesh, could have influenced their overall HRQoL. Our study also explored potential factors associated with these changes.

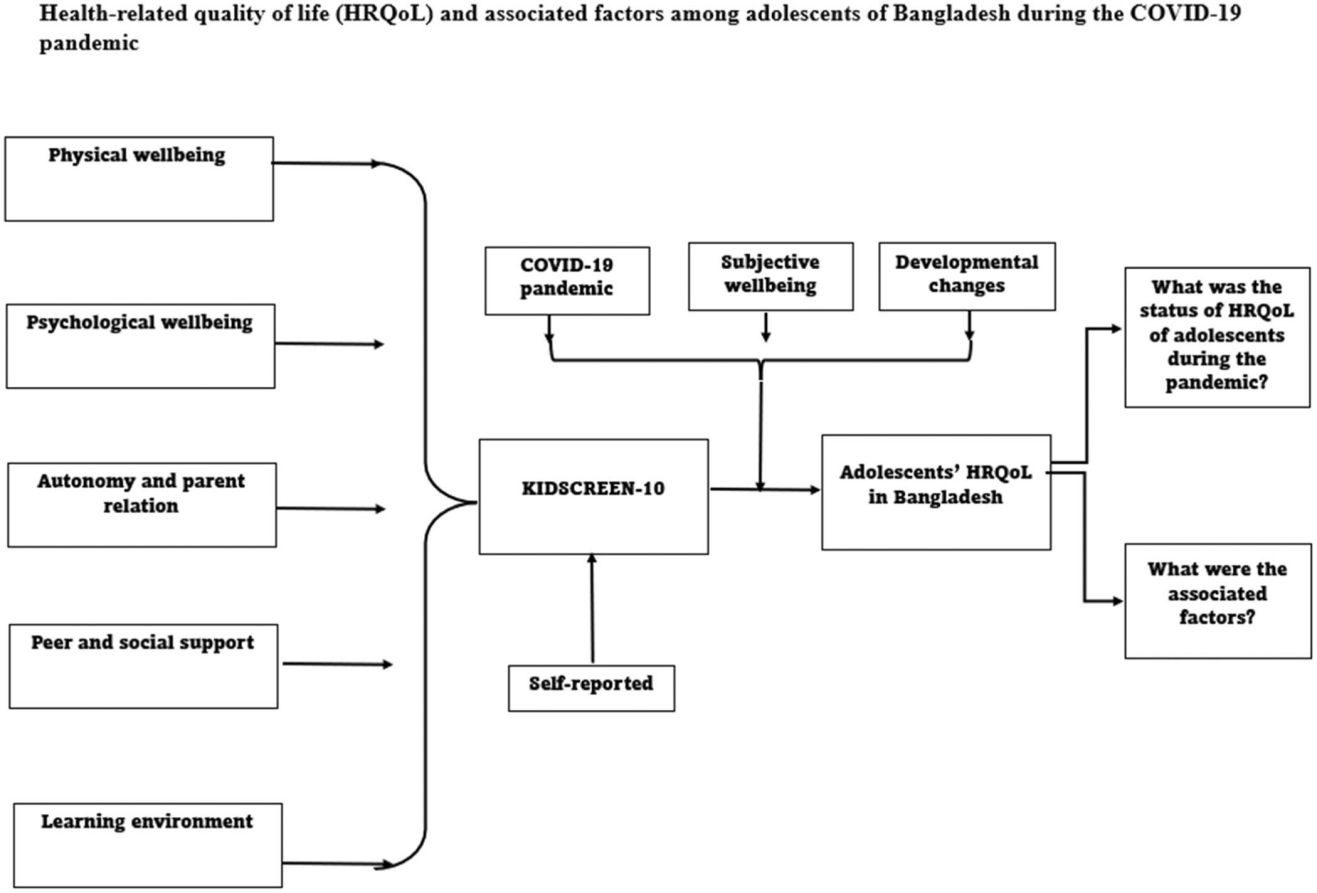

### 1.4 Bangladesh perspective

Bangladesh is one of the world's most populous countries, with adolescents constituting 20% of its 163 million population.^16^ HRQoL is a less explored issue in Bangladesh, particularly among adolescents living in the community, where only a few studies had been undertaken before the COVID‐19 pandemic.^17^, ^18^ Therefore, our primary objectives were to evaluate the HRQoL of adolescents in both urban and rural settings amid the COVID‐19 pandemic in Bangladesh, and to uncover the key determinants influencing the HRQoL of adolescents in the country.

## 2 METHODOLOGY

### 2.1 Study design and setting

This cross‐sectional study was conducted among the representative urban and rural adolescent populations of Bangladesh. There are eight administrative divisions consisting of 64 districts in Bangladesh. The sample was chosen from each division using two‐stage cluster sampling. For this study, one district was chosen randomly from each of the eight administrative divisions. These eight randomly selected districts were then divided into two clusters of four districts each. Then, urban and rural areas were chosen from these two clusters, with Sadar Upazila (subdistrict) and any other upazila other than Sadar Upazila being urban and rural regions, respectively. These upazilas served as the primary sample unit. In the case of urban areas, the secondary sampling unit of this study was a randomly selected ward of the municipal area of the Sadar Upazila. In the rural areas, the secondary sampling unit was a randomly selected village within a union of upazilas. Data were enumerated from secondary sampling units, and households from secondary sampling units were listed and mapped before data collection.

In this study, households were the ultimate sampling units. Before data collection, during preinterview screening, it was asked if any household members were between the ages of 14 and 19. Following that, a list of eligible households with data on the adolescent's age and gender was collected. Respondents who were absent or unwilling to participate were excluded from the study during data collection, and no proxy interviews were conducted.

### 2.2 Sample size

The estimated sample size was 234 based on a 13.6% prevalence of mental health disorders in children and adolescents,^19^ a 1.3 design effect, and a 5% margin of error. After adjusting for a 10.0% nonresponse rate, the final sample size was 257 × 8 = 2056, using administrative division as the cluster. After data cleansing, it was found that all 2030 records were complete and error‐free.

### 2.3 Data collection

During a 2‐week training session, 16 data collectors and eight field supervisors were taught how to collect data using Computer Assisted Personal Interviewing and REDCap (Research Electronic Data Capture) software. Furthermore, proper height (measured using a measuring tape), weight (NuLife Plus digital weight scale), and blood pressure (BP) (OMRON HEM‐7121) measures were instructed. During the measurement of height and weight, they were told to check that the participant had removed any watch or other heavy goods and was standing steady and looking forward.

A pretesting was undertaken in a nonsampling area before sending the data collectors into the field to ensure that the data collectors and study devices were effective.

During data collection, face‐to‐face interviews were conducted in an isolated place of the household of the adolescent. In coherence with ethical standards and cultural sensitivity, only female data collectors collected data from female respondents. Finally, manually collected height, weight and BP data were entered into the REDCap to complete data collection.

### 2.4 Outcome measure

In this study, the KIDSCREEN‐10, a tool for measuring HRQoL in adolescents, was used.^20^, ^21^, ^22^ Before using the scale, permission was taken from KIDSCREEN administrative group. The internal consistency of the items of this scale was found to be acceptable (Cronbach's alpha 0.74; significant positive correlation between items) in this study.

KIDSCREEN‐10 is a unidimensional tool that assesses physical well‐being, mood and emotion, autonomy, family and friends, and, finally, school environment over 1 week. Each of the aspects contained two items.^22^ It consists of 10 items and starts with “thinking of last week, have you … 1) felt fit and well, 2) felt full of energy, 3) felt sad, 4) felt lonely, 5) had enough time for yourself, 6) been able to do the things that you want in your free time, 7) parent(s) treated you fairly, 8) had fun with your friends, 9) got on well at school, 10) been able to pay attention at school?” There are five response categories for each item ranging from never to always or from not at all to extremely.^23^ This specific KIDSCREEN index has one extra question about the overall health status of the adolescents, which is excluded from the scoring of the scale.^24^

In Bangladesh, schools have been closed since March 16, 2020, although the government has ordered them to continue online education.^25^ So, in this study, issues pertaining to the school setting were asked regarding the adolescents' online educational environment.

For each of the response alternatives in this study, an operational definition was employed, such as 1–2 days from the previous week for the options “minimally and seldom,” 3–4 days for “quite often and moderately,” 5–6 days for “very and very often,” and 7 days for “always and extremely.” The response categories “not at all” and “never” were chosen when the option was not applicable to the respondent.

The scoring of the scale was done according to the manual provided by the KIDSCREEN group, converting the total score into a z‐score at first and then converting the z‐score to T score. Furthermore, when comparing the results of the current study, the reference value provided by the KIDSCREEN group was taken into account.

Finally, the mean KIDSCREEN T‐score was used to categorize the total T‐score. The T‐score mean was 50, so the scores 10–30 were regarded low, 31–50 moderate, while 50+ were rated high HRQoL. The T‐score was classified using the KIDSCREEN manual's ruler with answer category thresholds and t‐value distribution.^21^

### 2.5 Other measures

#### 2.5.1 Sociodemographic measures

Information on different sociodemographic variables was taken from adolescents, for example, age, sex, class/grade studying in, residing area, parental educational qualification, parental marital status, number of total siblings and family members. Then, certain variables were categorized according to pre‐existing categories and from previous literature, such as age was categorized into two groups, 14–16 years and 17–19 years, considering mid‐adolescence in one group and late adolescence in the other according to the guideline of WHO.^26^ Again, parental educational qualification was categorized and recategorized following previous studies.^27^, ^28^ Furthermore, The number of family members and number of siblings were then further categorized according to the ideal household size and total fertility rate of women in Bangladesh.^29^ Based on the information of household assets (mobile, electricity, fridge, table, chair, bed, car, motorcycle, and others), the wealth index was developed using principal component analysis.

#### 2.5.2 Short Warwick Edinburgh Mental Well‐being Scale (SWEMWS)

SWEMWS, a unidimensional 7‐item tool, was used in this study to enumerate adolescents' mental well‐being status. The license for the noncommercial use of the scale was taken before the study. This scale asks whether the respondent has been feeling optimistic about the future; useful; relaxed; dealing well with problems, thinking clearly, feeling close to other people; able to make up their mind about things over the last 2 weeks.^30^ On a 5‐point Likert scale, the response options ranged from 1 (none of the time) to 5 (all of the time). In line with the manual of the SWEMWS, the raw scores were summed at first and then converted into a total metric score using the SWEMWS conversion table.^31^ After conversion, the SWEMWBS score ranged from 7 to 35.^32^ Then, the total score of the SWEMWS was categorized into three categories, where the range of low mental well‐being was 7.0–19.3 and the ranges of medium and high mental well‐being were 20.0–27.0 and 28.1–35.0, respectively.^33^ The value of Cronbach's alpha for SWEMWS in this study was 0.74.

### 2.6 Athens Insomnia Scale (AIS)

For measuring the sleep patterns of adolescents as well as the impact of sleep patterns on physical health, the self‐reported AIS was used in this study. Based on ICD‐10 diagnostic criteria, this scale was developed to enumerate the severity of insomnia.^34^ There are eight items on the scale. The first five items assess difficulty with sleep induction, awakening during the night, early morning awakening, total sleep time, and overall sleep quality. The last three items assess the well‐being, overall functioning and sleepiness during the day.^35^ Generally, the respondents are asked about their sleep patterns in the last month and if those happened to them at least three times a week. Each item of AIS is rated from 0 to 3, while 0 indicates no problem at all and three indicates a very serious problem. The primary validation of the scale demonstrates a high Cronbach's alpha (0.75–0.90) and the test–retest reliability over 1 week was 0.90.^35^ The Cronbach's alpha for AIS, found in this study was 0.90.

This study used proper Bangla translation of the scale, and the operational definition was set for each option. The instruction for 1 month for this scale was converted into 30 days in this study. The options were changed accordingly; for example, for the options “Slightly delayed” or “Slightly insufficient,” the day limit was 10–15 days and for the options “Markedly delayed” or “Markedly insufficient,” the day limit was 15–25 days. More than 25 days was the day limit for the options “Very delayed or did not sleep at all” or “Very insufficient or did not sleep at all.” The total scores range from 0 to 24 and higher scores indicate serious insomnia symptoms. According to the total score, insomnia was then categorized into 0–5 = no insomnia, 6–9 = suspected insomnia, and ≥10 = definite insomnia.^36^

#### 2.6.1 Alabama Parenting Questionnaire (APQ)

This study used a short form of the APQ, which studies three dimensions of parenting behavior: positive parenting, inconsistent discipline, and poor supervision. There are nine questions in the scale in total and three questions for each dimension.^37^ The response options range from 1 (never) to 5 (always) on the Likert scale. The scale has been widely used and found reliable and valid.^38^ The internal consistency for APQ in this study was found to be 0.68. Though this scale is applicable to parents, in this study, it was adapted for adolescents only. The items were adapted for adolescents; for example, “You threaten to punish your child and then do not actually punish him/her” (inconsistent discipline) was adapted as “Your parents threaten to punish you and then do not actually punish you.” By adding the scores of each dimension (positive parenting [items 1,6,7], inconsistent discipline [items 2,4,9], and poor supervision [items 3,5,8]) total score for APQ was obtained.

#### 2.6.2 Food insecurity index

To measure food insecurity among adolescents, The Food Insecurity Experience Scale was used, developed in 2014 by the UN FAO—Voices of the Hungry project to measure food insecurity.^39^ The scale enquires about the self‐reported experience of food insecurity due to lack of money and other resources over a 12‐month recall period and disregards the frequency of occurrence. Based on the total score, food insecurity can be classified into three categories: (1) food security with raw scores = 0–3; (2) moderate food insecurity, with raw scores = 4–6; and (3) Severe food insecurity, with raw scores = 7–8.^40^ The value of Cronbach's alpha for this scale in this study was 0.88.

#### 2.6.3 Depression, Anxiety, Stress Scale‐21 (DASS‐21)

DASS‐21 was used to find out the severity of depression, anxiety, and stress among adolescents. DASS‐21 is the shorter form of the previously established scale DASS‐42.^41^ There are three subparts of the scale (depression, anxiety, and stress), consisting of seven items in each subscale. Because of its easy‐to‐administer nature, this scale has been widely used in clinical and nonclinical settings to assess depression, anxiety, and stress in the most recent week. We used the Bangla‐validated DASS‐21.^42^ Answers are reported on a 4‐point Likert scale and the range is 0–3. 0 indicates “Did not apply to me at all,” 1 indicates “Applied to me to some degree, or some of the time,” while 2 indicates “Applied to me to a considerable degree, or a good part of the time,” and 3 indicates “Applied to me very much, or most of the time.”

In this study, an operational definition was set for each option, for example, if the respondent states that it is applicable for him/her for 1–3 days of the last 7 days, then the option “Applied to me to some degree, or some of the time” was appropriate for him/her. Likewise, for 4–5 days the option “Applied to me to a considerable degree, or a good part of time” and 6–7 days the option “Applied to me very much, or most of the time” were applied for the respondent. If the question is not applied for him at all, then the option “Did not apply to me at all” was put for the respondent. In this study, Cronbach's alpha for this scale was found 0.88.

The total scores for this questionnaire are obtained according to subscales: depression, anxiety, and stress. The summed numbers in each subscale are multiplied by 2. According to the total score, the subscales were categorized into normal, mild, moderate, severe, and extremely severe.

#### 2.6.4 Brief Resilient Coping Scale (BRCS)

In this study, the BRCS was used to evaluate the resilient coping status of adolescents. There are four items in the scale: (1) I look for creative ways to alter difficult situations, (2) regardless of what happens to me, I believe I can control my reaction to it, (3) I believe that I can grow in positive ways by dealing with difficult situations, (4) I actively look for ways to replace the losses I encounter in life.^43^ The scoring of the scale is done by a 5‐point Likert scale ranging from 1(does not describe me at all) to 5 (describes me very well) and the total score range from 4 to 20.^44^, ^45^ Based on the total score of the BRCS, the respondents were then categorized into low, medium and high resilient copers, where the total score range for low, medium and high resilient copers were 4–13 points, 14–16 points, and 17–20 points, respectively.^43^ Cronbach's alpha of BRCS found in previous studies were 0.76 and 0.68.^44^, ^45^ In this study, Cronbach's alpha was 0.85.

#### 2.6.5 Screen‐based activities

Information was taken on adolescent's screen‐based activities, such as whether they were using the internet at the time of data collection or not. If the respondents provided an affirmative response, they were asked how many hours they used the internet per day and week. Then, the total hours of internet use in a week were categorized into <15 h per week and ≥15 h per week based on previous research.^46^

#### 2.6.6 Body mass index (BMI)

Height and weight were collected during data collection and then from those data, BMI was calculated using the formula kg/m^2^. The classification for South Asian adolescents was used in this study. The cut‐off points were: <18.5 kg/m^2^—underweight, 18.5–22.9 kg/m^2^—normal weight, 23–24.99 kg/m^2^—overweight, and ≥25 kg/m^2^—obese.^47^, ^48^, ^49^ Considering these cut‐offs, the BMIs of the adolescents were classified.

### 2.7 Statistical analysis

The variables were categorized into sociodemographic variables, food insecurity and anthropometry, parenting style, lifestyle, and mental health for statistical analysis. Respondent's sex, age, the total number of family members and the total number of siblings, parental educational level, residential area and wealth index were put under sociodemographic variables. Under the category of food insecurity and anthropometry food insecurity, BMI and BP were put. Positive parenting, inconsistent parenting discipline and poor parental supervision were the categories of the category of parenting style. In the category lifestyle, insomnia, screen‐based activities, such as internet use and total internet usage hour per week, were included. In the fifth category, mental health, mental well‐being, depression, anxiety and stress, and resilient coping were included.

Relationships between variables were evaluated by Pearson correlation along with the chi‐square test, Fisher's exact test, as well as hierarchical multilinear regression analysis. Independent variables found significant in the univariate analysis were further analyzed using hierarchical multiple regression models. In the first stage, sociodemographic variables were entered into Block 1, and then in second Block food insecurity was entered. In Block 3, parenting styles were entered and in Block 4 and Block 5 lifestyle and mental health variables were entered, respectively. There were no missing data for any independent and dependent variables. All types of univariate/multiple normality, linearity, homoscedasticity, and diagnostic testing for multicollinearity and independence of errors were checked before running the analysis. The significance level in this study was *p *< 0.05. All the data were analyzed using Statistical Package for the Social Sciences software for Windows, version 25.0.

### 2.8 Ethical considerations

Ethical clearance was taken from the Institutional Review Board of Bangabandhu Sheikh Mujib Medical University (Memo no: BSMMU/2021/4408). Besides, before data collection, informed written consent and assent were taken from adolescents and their parents in front of witnesses.

## 3 RESULT

Among the respondents, 4.8% of the adolescents were found to have poor HRQoL, while 47.4% and 47.8% were found to have moderate and high HRQoL, respectively. This study found the minimum value of the T score was 10.81, while the maximum value was 74.01.

**Table 1:** Association between HRQoL and sociodemographic variables, food insecurity, anthropometry.

Variable	(%)	Low HRQoL^a^	Moderate HRQoL^a^	High HRQoL^a^	*p* Value
Sociodemographic variables					
Sex					
Male	39.3	28 (29.2)	300 (31.2)	469 (48.3)	<0.001^b^
Female	60.7	68 (70.8)	663 (68.8)	502 (51.7)	
Age					
14–16	76.4	53 (55.2)	714 (74.1)	783 (80.6)	<0.001^b^
17–19	23.6	43 (44.8)	249 (25.9)	188 (19.4)	
Total number of siblings					
≤2 siblings	44.1	44 (45.8)	382 (39.7)	469 (48.3)	0.002^c^,^b^
3–6 siblings	54.8	50 (52.1)	570 (59.2)	493 (50.8)	
>6 siblings	1.1	2 (2.1)	11 (1.1)	9 (0.9)	
Total number of family members					
1–4 members	42.3	40 (41.7)	388 (40.3)	430 (44.3)	0.510^c^
5–8 members	54.7	54 (56.3)	544 (56.5)	512 (52.7)	
>8 members	3.1	2 (2.1)	31 (3.2)	29 (3.0)	
Father's education					
<Grade XII	84.1	78 (81.3)	833 (86.5)	797 (82.1)	0.025^d^
≥Grade XII	15.9	18 (18.8)	130 (13.5)	174 (17.9)	
Mother's education					
<Grade XII	90.3	86 (89.6)	885 (91.9)	863 (88.9)	0.078
≥Grade XII	9.7	10 (10.4)	78 (8.1)	108 (11.1)	
Residential area					
Urban	50.4	77 (80.2)	470 (48.8)	477 (49.1)	<0.001^b^
Rural	49.6	19 (19.8)	493 (51.2)	494 (50.9)	
Wealth index					
Low	33.9	31 (32.3)	357 (37.1)	300 (30.9)	0.017^d^
Middle	25.3	20 (20.8)	249 (25.9)	244 (25.1)	
High	40.8	45 (46.9)	357 (37.1)	427 (44.0)	
Food insecurity and anthropometry					
Food insecurity					
Secure	77.8	50 (52.1%)	678 (70.4)	851 (87.6)	<0.001^b^
Moderate insecure	14.5	24 (25.0)	192 (19.9)	78 (8.0)	
Severe insecure	7.7	22 (22.9)	93 (9.7)	42 (4.3)	
BMI					
Underweight	45.1	46 (47.9)	437 (45.4)	432 (44.5)	0.993^c^
Normal weight	38.7	36 (37.5)	371 (38.5)	378 (38.9)	
Overweight	7.2	5 (5.2)	69 (7.2)	72 (7.4)	
Obese	9.1	9 (9.4)	86 (8.9)	89 (9.2)	

Abbreviations: BMI, body mass index; HRQoL, health‐related quality of life.

^a^ Figure within parenthesis denoted corresponding column percentage.

^b^ Significant at 0.01 level.

^c^ Fisher's exact test.

^d^ Significant at 0.05 level.

The association between HRQoL and sociodemographic variables as well as food insecurity and anthropometry, have been presented in Table 1. Sex (*p *< 0.001) and age (*p* < 0.001) were found to have a significant association with HRQoL. Again, a total number of siblings (*p* = 0.002), residential area (*p *< 0.001) and food insecurity (*p* < 0.001) were found to have a strong significant association with HRQoL. Besides, father's education (*p* = 0.025) and wealth index (*p* = 0.017) also had a significant relationship with HRQoL.

Table 2 shows that total internet usage time in a week (*p* < 0.001) was found to have a significant association with HRQoL among lifestyle‐related factors. Among mental health‐related factors, mental well‐being status (*p* < 0.001), anxiety (*p* < 0.001) and resilient coping (*p* < 0.001) were found to have significant associations with HRQoL.

**Table 2:** Association between HRQoL and lifestyle and mental health variables.

Variable	%	Low HRQoL^a^	Moderate HRQoL^a^	High HRQoL^a^	*p* Value
Lifestyle variables					
Insomnia					
No insomnia	81.9	84 (87.5)	808 (83.9)	771 (79.4)	0.087^b^
Suspected insomnia	12.6	8 (8.3)	108 (11.2)	139 (14.3)	
Definite insomnia	5.5	4 (4.2)	47 (4.9)	61 (6.3)	
Screen‐based activities					
Currently using internet					
No	47.9	13 (21.3)	79 (17.8)	104 (18.8)	0.798
Yes	52.1	48 (78.7)	365 (82.2)	449 (81.2)	
Total internet usage time in a week					
<15 h per week	9.8	28 (29.2)	91 (9.4)	80 (8.2)	<0.001^c^
≥15 h per week	90.2	68 (70.8)	872 (90.6)	891 (91.8)	
Mental health variables					
Mental well‐being					
Poor mental well‐being	29.6	65 (67.7)	439 (45.6)	96 (9.9)	<0.001^c^
Good mental well‐being	70.4	31 (32.3)	524 (54.4)	875 (90.1)	
Depression					
No	82.2	77 (80.2)	786 (81.6)	806 (83.0)	0.649
Yes	17.8	19 (19.8)	177 (18.4)	165 (17.0)	
Anxiety					
No	86.2	49 (51.0)	774 (80.4)	927 (95.5)	<0.001^c^
Yes	13.8	47 (49.0)	189 (19.6)	44 (4.5)	
Stress					
No	84.5	84 (87.5)	832 (86.4)	799 (82.3)	0.030^d^
Yes	15.5	12 (12.5)	131 (13.6)	172 (17.7)	
Resilient coping					
Low resilient copers	16.6	45 (46.9)	206 (21.4)	85 (8.8)	<0.001^c^
Medium resilient copers	50.6	35 (36.5)	490 (50.9)	502 (51.7)	
High resilient copers	32.9	16 (16.7)	267 (27.7)	384 (39.5)	

Abbreviation: HRQoL, health‐related quality of life.

^a^ Figure within parenthesis denoted corresponding column percentage.

^b^ Fisher's exact test.

^c^ Significant at 0.01 level.

^d^ Significant at 0.05 level.

Multiple hierarchical linear regression using HRQoL as the dependent variable and other predictive variables have been demonstrated in Table 3. Data of the final model (except R^2^, R^2^ change, and F change) are presented in this table. In model 1, among the sociodemographic variables, age (B: −0.671; *β*: −0.089; *p* < 0.001) and number of total siblings (B: −0.316; *β*: ‐0.036; *p *= 0.034) were found to have negative significant association with HRQoL. This model explained 7.8% of the variance with HRQoL score, which was statistically significant with F = 28.373 (*p *< 0.001). Model 2 (Food insecurity) explained 5.0% of the variance with HRQoL score, which was statistically significant with F = 116.490 (*p* < 0.001) and food insecurity (B: −2.010; *β*: −0.084; *p* < 0.001) had a significant negative association with HRQoL, implying deterioration of HRQoL with the increase in food insecurity. In model 3, positive parenting (B: 0.409; *β*: 0.090; *p* < 0.001) and inconsistent parenting discipline (B: 0.266; *β*: 0.087; *p* < 0.001) were found to have a significant positive association with HRQoL and the model was found significant with F = 57.546 (*p* < 0.001). Model 4 was significant with F = 7.020 (*p* < 0.001) and among the lifestyle variables, internet use ≥15 h per week (B: 1.478; *β*: 0.044; *p* = 0.015) had a significant positive association with HRQoL. In model 5, variables of mental health explained 23.7% of the variance in HRQoL score, which was statistically significant with F = 170.299 (*p* < 0.001). Good mental well‐being (B: 5.662; *β*: 0.258; *p* < 0.001) and resilient coping (B: 0.306; *β*: 0.100; *p* < 0.001) had a significant association with HRQoL, while depression (B: −0.321; *β*: −0.175; *p* < 0.001), anxiety (B: −0.362; *β*: −0.161; *p* < 0.001) and stress (B: −0.150; *β*: −0.098; *p* < 0.001) had a significant negative association with HRQoL.

**Table 3:** Hierarchical multiple regression analysis predicting the factors associated with HRQoL among adolescents during COVID‐19.

Variable	Final model				R^2^	R^2^ change	F change
	B	95% CI for B	*β*	*p* Value			
Sociodemographic variables							
Sex					0.078	0.078	28.373^a^
Female [ref: male]	−0.314	−1.038 to 0.410	−0.015	0.395			
Age (year)	−0.671	−0.930 to −0.413	−0.089	<0.001			
No. of total siblings	−0.316	−0.608 to −0.024	−0.036	0.034			
Mother's education							
<XII grade [ref: ≥XII grade]	0.116	−1.036 to 1.268	0.003	0.844			
Parental marital status							
Currently not married [ref: currently married]	0.122	−0.996 to 1.240	0.004	0.831			
Residential area							
Urban area [ref: rural area]	−0.367	−1.074 to 0.340	−0.018	0.309			
Food insecurity							
Food insecure [ref: food secure]	−2.010	−2.868 to −1.153	−0.084	<0.001	0.128	0.050	116.490^a^
Parenting style							
Positive parenting	0.409	0.248–0.571	0.090	<0.001	0.197	0.069	57.546^a^
Inconsistent parenting	0.266	0.155–0.378	0.087	<0.001			
Poor parenting	0.114	−0.010 to 0.237	0.033	0.071			
Lifestyle							
Insomnia [ref: no insomnia]	0.575	−0.280 to 1.430	0.022	0.187	0.205	0.008	7.020^a^
Currently using internet							
Yes [ref: no]	0.573	−0.166 to 1.312	0.029	0.129			
Total internet use in a week							
≥15 h per week [ref: <15 hours/week]	1.478	0.286–2.671	0.044	0.015			
Mental health							
Mental well‐being					0.441	0.237	170.299^a^
Good [ref: poor mental well‐being]	5.662	4.836−6.489	0.258	<0.001			
Depression score	−0.321	−0.414 to −0.229	−0.175	<0.001			
Anxiety score	−0.362	−0.458–0.265	−0.161	<0.001			
Stress score	−0.150	−0.223–0.077	−0.098	<0.001			
Resilient coping	0.306	0.192–0.421	0.100	<0.001			

*Note*: B, unstandardized coefficient; *β*, standardized coefficient; R^2^, coefficient of multiple determination. Currently not married: separated, divorced, widowed.

Abbreviations: CI, confidence interval; HRQoL, health‐related quality of life.

^a^ F change significant at 0.01 level.

## 4 DISCUSSION

### 4.1 Adolescents' HRQoL during the pandemic in Bangladesh

To our knowledge, this is the first population‐based study that explores HRQoL and its associated factors among adolescents during the COVID‐19 pandemic in Bangladesh. The maximum t‐value found in this study is consistent with the individual norm data from the United Kingdom for the KIDSCREEN‐10, where the T score for adolescents aged 12–18 was 71.2.^21^ This study also found that the mean of the KIDSCREEN‐10 score was 36.7, meaning that most adolescents were experiencing moderate HRQoL at the time of data collection. This finding is different from the systematic review of Nobari et al., which stated that during the COVID‐19 pandemic among children and adolescents, a reduction of HRQoL has been observed.^50^ Besides, Ravens‐Sieberer et al. reported that during the COVID‐19 pandemic, the HRQoL of adolescents in Germany was significantly lower.^9^ Supporting this finding, Riiser et al. also affirmed that adolescents were experiencing lower HRQoL during the COVID‐19 pandemic compared to any other time.^23^ The difference in the low HRQoL of adolescents in other studies and moderate HRQoL in the present study might be due to various reasons. For example, the studies that found significantly lower HRQoL among adolescents were conducted in the initial phase of the pandemic when the lockdown was strictly imposed since the situation was unique and worsening daily.

Along with these, adolescents were experiencing social distancing for the first time in their lives, which initiated uncertainty, stress and other mental and somatic symptoms, which had a principal role in reducing the HRQoL of adolescents.^51^, ^52^, ^53^ On the contrary, the current study was conducted when the adolescents had become used to the pandemic after more than 1 year and the lockdown, social distancing were relaxed and most importantly, the adolescents had the chance to meet with their peers while going to submitting their scheduled home task at schools once or twice in a month. However, more explorative studies are suggested to find out the difference between the current study and other studies.

### 4.2 Factors associated with adolescents' HRQoL in Bangladesh during the pandemic

In this study, age was found to have a significant negative relationship with HRQoL, implying that with the increase of age, HRQoL decreases among adolescents during COVID‐19. Though no significant association was found between adolescents' sex and HRQoL, previous studies have said that with the increase of age, female adolescents become more vulnerable to experiencing low HRQoL compared to their male counterparts.^9^ Moreover, as adolescents age, they become increasingly susceptible to real‐life issues related to low socioeconomic status, parental unemployment, heightened academic pressure, and other factors that can significantly affect their HRQoL.^9^ This study also found a significant negative relationship with the number of total siblings, suggesting with the increasing number of siblings, adolescents' HRQoL decreases. Mutual understanding and good relationships between siblings have a prominent impact on an individual's life and they spend most of their time together, which builds companionship among siblings.^54^, ^55^ Conversely, when there are many siblings, there is a chance of chaos and other socioeconomic problems, such as food insecurity and inaccessibility to education.^4^

Food insecurity had a significant inverse relationship with the HRQoL of adolescents in this study. This finding is supported by Casey et al., which revealed a significant association between food insecurity and low HRQoL.^56^ Studies have reported that compared to adolescents with food insecurity, adolescents who have food insecurity have lower general health status and more negative symptoms.^57^, ^58^ Though there are no established reasons regarding the association between food insecurity and lower HRQoL, Kihlstrom et al. stated that when there is insufficiency in the food supply in the family, it may affect both the physical and mental health of people in many aspects, thereby affect the QOL of people.^59^ Gassman‐Pines et al. mentioned in their study that during socioeconomic disasters such as a pandemic, it is likely that parents will lose jobs, leading to a decrease or complete loss of household income, which can give rise to various problems including food insecurity.

They also stated that parents might go through severe stress during this time, which has an effect on the mental health of adolescents.^60^ Surprisingly, this study found a significant positive association between inconsistent parenting discipline and HRQoL and the same with the case with positive parenting style. Previous studies have pointed out that during the COVID‐19 pandemic, parental negative affect has been increased, which may impact the perceived well‐being of adolescents during this critical time.^61^ Parental joblessness and income loss may initiate depressive symptoms and stress among parents and parental mental health problems have an association with the mental health condition of adolescents.^62^ Different studies showed that several factors are associated with parental mental health during COVID‐19, for example, being a single parent, parenting younger children and adolescents, financial hardship, and so on.^63^, ^64^ The current study found an association between mental health‐related factors and the HRQoL of adolescents. Good mental well‐being and resilient coping of adolescents have been found to have a significant positive association with HRQoL of adolescents, while depression, anxiety, and stress had a significant negative association with adolescents' HRQoL. During the pandemic, social isolation may trigger aggressiveness and other emotional pain, while lack of social interaction may impact the feeling of vitality through emotional perception, which ultimately affects the perception of general health. The combination of social isolation and social interaction has a direct effect on the mental health of adolescents and the analogous reaction has the ability to cause harm to physical health. Consequently, the body functionality decreases and perception of physical pain increases, thus, the HRQoL lowers during the pandemic.^65^ More longitudinal studies are needed to establish a more causal relationship between the pandemic and mental health and the impact on HRQoL.

### 4.3 Implication of study findings

This study examines the scenario of adolescent HRQoL during COVID‐19 in Bangladesh and identifies the factors associated with it. Given the scarcity of research on adolescents' HRQoL in low and middle‐income countries (LMICs), specially in Bangladesh, these findings are of significant importance. Therefore, the findings of this study will serve as baseline evidence for future studies on this topic for adolescents in Bangladesh as well as other LMICs. Additionally, based on the findings of the current study, future large‐scale studies on adolescents' HRQoL following different emerging research methods, such as online photovoice, can be designed and implemented in LMICs.

### 4.4 Limitations of the study

Despite being the first population‐based study to enumerate HRQoL and associated factors of adolescents during the COVID‐19 pandemic in Bangladesh, there are some limitations to the study. First, the cross‐sectional study design was unable to establish a causal relationship with the factors found associated with adolescents' HRQoL. Second, data collection from adolescents always involves the risk of recall bias as well as response bias due to misinterpretation of the terminology. Third, there might be a type 1 error in the study since multiple statistical testing had been done but statistical correction of *p* values was not conducted in the study. Therefore, extensive research is needed on this subject with representative data which will be free from any type of error. Fourth, we used several psychometrics that were not culturally validated in Bangla. Fifth, DASS‐21 is not a diagnostic tool for depression, anxiety, and stress. Furthermore, there was no assessment of validity for Bangla DASS‐21 among adolescents.

## 5 CONCLUSION

Adolescents of Bangladesh were found to have moderate HRQoL mostly during the COVID‐19 pandemic. Increasing age, food insecurity, mental health‐related factors were found to have a significant impact on adolescents during the pandemic. Given the limited prior research on this critical topic, the findings of this study can act as a baseline for future interventional studies. It is anticipated that such interventions will lead to substantial improvements in postpandemic Bangladesh, ultimately contributing to the achievement of the Sustainable Development Goals within the stipulated time frame.

## AUTHOR CONTRIBUTIONS

**Afifa Anjum**: Conceptualization; data curation; formal analysis; project administration; validation; writing—original draft; writing—review and editing. **Sabrina Mousum**: Conceptualization; data curation; formal analysis; project administration; validation; writing—original draft; writing—review and editing. **Zubair Ahmed Ratan**: Validation; writing—original draft; writing—review and editing. **Marium Salwa**: Conceptualization; formal analysis; validation; writing—original draft; writing—review and editing. **Md Maruf H. Khan**: Project administration; validation; writing—review and editing. **Mohammad Tanvir Islam**: Conceptualization; validation; writing—review and editing. **S. M. Yasir Arafat**: Validation; writing—original draft; writing—review and editing. **M. Atiqul Haque**: Conceptualization; formal analysis; funding acquisition; supervision; validation; writing—original draft; writing—review and editing.

## CONFLICT OF INTEREST STATEMENT

S. M. Yasir Arafat is an Editorial Board member of Health Science Reports and co‐author of this article. They were excluded from editorial decision‐making related to the acceptance of this article for publication in the journal. The remaining authors declare no conflict of interest.

## TRANSPARENCY STATEMENT

The lead author M. Atiqul Haque affirms that this manuscript is an honest, accurate, and transparent account of the study being reported; that no important aspects of the study have been omitted; and that any discrepancies from the study as planned (and, if relevant, registered) have been explained.

## Data Availability

The data supporting this study's findings are available on request from the corresponding author.
